# Phytochemical, antioxidant, and antimicrobial properties of *Bellardia trixago* methanol and ethanol extracts: insights from ADMET and molecular docking approaches

**DOI:** 10.1007/s13197-025-06217-y

**Published:** 2025-01-27

**Authors:** Erdi Can Aytar, Betül Aydın, Alper Durmaz, Emine Incilay Torunoğlu

**Affiliations:** 1 Faculty of Agriculture, Department of Horticulture, Usak University, Uşak, Türkiye; 2 Faculty of Science, Department of Biology, Gazi University, Ankara, Türkiye; 3 Ali Nihat Gokyigit Botanical Garden Application and Research Center, Artvin Coruh University, Artvin, Türkiye; 4 Faculty of Medicine, Department of Medical Biochemistry, Necmettin Erbakan University, Konya, 42090 Türkiye

**Keywords:** ADMET, Antimicrobial activity, *Bellardia trixago*, Chemical composition, Molecular docking, Phytochemical screening

## Abstract

This study investigates the antioxidant, antimicrobial, and phytochemical properties of ethanol and methanol extracts derived from *Bellardia trixago* flowers alongside molecular docking and pharmacokinetic assessments of stigmasterol, a key bioactive compound. The antioxidant activities of the ethanol and methanol extracts were determined, with the methanol extract demonstrating higher activity, 0.370 ± 0.002 mg/mL, compared to the ethanol extract, 0.95 ± 0.09 mg/mL. The total phenolic content of the ethanol extract was 79.14 ± 2.30 mg GAE/g extract DW, and its flavonoid content was 244.31 ± 12.51 mg QE/g extract DW. The methanol extract contained a lower phenolic content of 51.50 ± 1.43 mg GAE/g extract DW but a higher flavonoid content of 251.67 ± 6.68 mg QE/g extract DW. The ethanol extract exhibited a superior iron chelation capacity of 8.21 ± 0.09 mg/mL relative to the methanol extract of 6.68 ± 0.28 mg/mL. Antimicrobial assays demonstrated that both extracts exhibited strong bactericidal and bacteriostatic effects, with MIC values ranging from < 0.39 mg/mL to > 125 mg/mL. The highest antimicrobial activity was observed against *Bacillus cereus* NRRL B-3711. Phytochemical analysis identified 25 compounds in the methanol extract and 20 in the ethanol extract. Stigmasterol was the major constituent in both extracts, accounting for 26.51% in ethanol and 12.65% in methanol. Molecular docking studies of stigmasterol revealed strong binding affinities with several protein targets, including *Candida albicans* Complex III2 (-8.7 kcal/mol), *Escherichia coli* HipBST (-7.2 kcal/mol), and the ribosomal subunit of *Staphylococcus aureus* (-7.0 kcal/mol). These interactions highlight stigmasterol’s potential role in modulating bacterial and fungal protein functions, emphasising the potential therapeutic applications of *B. trixago*, particularly in antimicrobial and antioxidant contexts.

**Supplementary Information:**

The online version contains supplementary material available at 10.1007/s13197-025-06217-y.

## Introduction

*Bellardia trixago* (L.) All. (syn. *Bartsia trixago* L.) is an herbaceous plant from the Orobanchaceae, found in the Mediterranean region, spanning from Portugal to Türkiye (Echeverría et al. 2020). Ethnobotanically, the flowers of *B. trixago* are consumed as food; however, research on its biological effects and medicinl potential is quite limited (Formisano et al. 2008; Soriano et al. 2022).

Secondary metabolites play an important role as part of the natural defence mechanisms of plants and have been used in traditional medicine for a long time. These compounds facilitate the extraction of various active substances for pharmacological purposes and the production of herbal medicines. *B. trixago* is an ethnomedicinal plant used by local populations worldwide for various therapeutic purposes (Yarazari and Jayaraj 2022).The existing literature on the essential oils of *B. trixago* is limited. A study by (Formisano et al. 2008) identified the major components of *B. trixago* essential oil as (E, E)-farnesyl acetone, trixagol, and 4-vinyl guaiacol. In another study by (Semiz and Günal 2023), cembrene was identified as the main component, with phellandral and α-terpineol as the other predominant components. Additionally, a study conducted by (Esteban et al. 1996) utilised automatic thermal desorption techniques to determine the volatile components of some aromatic plants, revealing that trixagoyl acetate, trixagol, and trixagoene were the three most abundant volatile organic compounds in *B. trixago*. The applications of essential oils in medical, cosmetic, and industrial fields offer potential advantages when considering their pharmacological properties. These studies provide an important foundation for discovering new antibacterial treatment methods.

Plant-based antioxidants are critical in maintaining cellular redox homeostasis by catalysing or neutralising free radicals (Joshi et al. 2005; Kuczyńska et al. 2022). Free radicals, particularly reactive oxygen species (ROS) and reactive nitrogen species (RNS), are molecules with free electrons, making them highly reactive. Among the main types of ROS are hydroxyl radicals, superoxide anion, nitric oxide, and peroxyl radicals; there are non-radical species like hydrogen peroxide and singlet oxygen (Fubini and Hubbard 2003). ROS can initiate oxidative stress in cells by causing oxidative damage to proteins, lipids, DNA, and other vital biomolecules. This oxidative stress plays a role in the pathogenesis of numerous metabolic disorders, including aging, neurodegenerative diseases, and proliferative cell condition (Rathod et al. 2022).

Antioxidants protect cellular components by neutralising the harmful effects of ROS and by blocking or interrupting the chain reactions they initiate (Walker et al. 2020). Flavonoids, polyphenolic compounds widely distributed in the plant kingdom, are particularly noted for their antioxidant activity (Hemmami et al. 2023). Consequently, the potential application of plant-based antioxidants as alternative therapeutic agents underscores the need for more extensive global research (Jagwani et al. 2020).

While the beneficial health effects of plant-based antioxidants are widely researched, their antimicrobial properties have also garnered attention. However, the development of bacterial resistance to conventional antibiotics has become a major global health concern. The antimicrobial resistance crisis has been exacerbated by the excessive and improper use of antibiotics, leading to the emergence of resistant strains such as methicillin-resistant *Staphylococcus aureus*, vancomycin-resistant enterococci, drug-resistant *Streptococcus pneumoniae*, and *Mycobacterium tuberculosis*. Similarly, fungal pathogens have developed resistance to polyenes, azoles, and echinocandins, with resistant strains reported across various fungal species (Robbins et al. 2017; Aslam et al. 2018; Aleksic Sabo and Knezevic 2019).

The primary objective of this study is to investigate the antioxidant and antimicrobial properties of *B. trixago* flower extracts, comparing the efficacy of ethanol and methanol extracts. Additionally, the study aims to identify and characterise the bioactive compounds in these extracts, focusing on stigmasterol, and assess its potential pharmacological applications through molecular docking studies against key protein targets in bacterial and fungal systems. Furthermore, the research evaluates stigmasterol’s pharmacokinetic properties and safety profile, contributing to understanding its therapeutic potential and suitability for future medical applications.

## Materials and methods

### Plant material

**Fig. 1:**
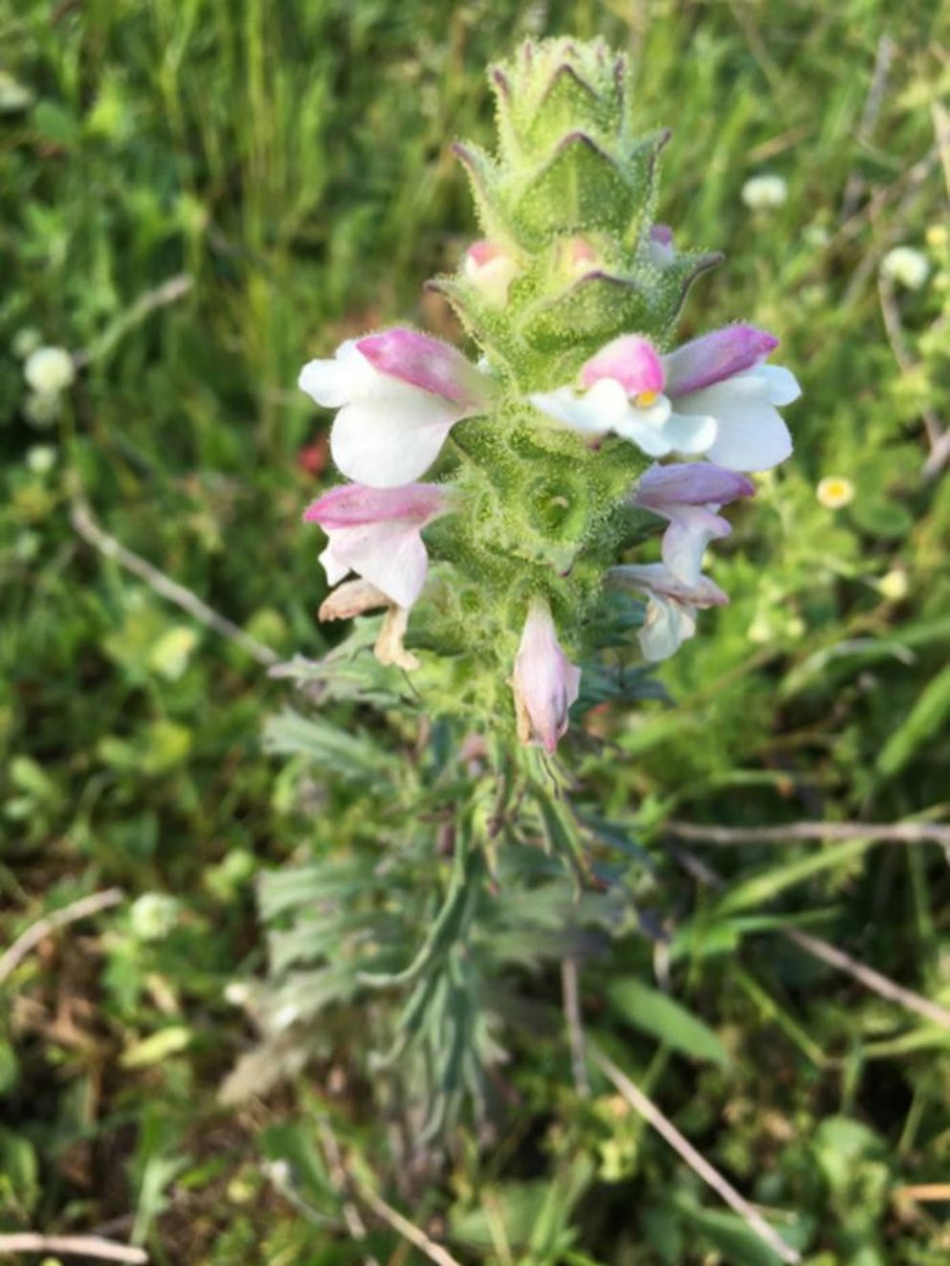
Flowering branch of *Bellardia trixago*

**Fig. 2:**
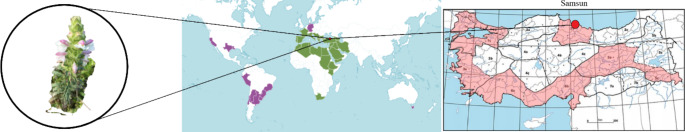
Collection and distribution area of *Bellardia trixago*

*Bellardia trixago* plant samples were collected in May 2021 from two locations: the forest and grassland area behind the rectorate building at Ondokuz Mayıs University (41° 21’ 50’’ N 36° 11’ 51’’ E) and the valley slopes behind the Samsun Metropolitan Municipality equestrian sports facilities (41° 21’ 23’’ N 36° 11’ 14’’ E). Figure 1 shows the flowering branch and Fig. 2 collection area of *B. trixago*. The collection followed national and international regulations, including IUCN and CITES guidelines. Dr. Alper DURMAZ identified the specimens preserved in the Ondokuz Mayıs University Herbarium (OMUB) under accession number 8342. The samples were dried at 50 °C for 48 h, ground using a mixer and stored in sealed jars to prevent air exposure.

### Plant extraction

The freshly harvested plants were washed with distilled water and cleaned, with the flower parts being separated. The separated flowers were dried in an oven at 40 °C for two days. After drying, the flowers were ground into a fine powder using a blender. 200 g of dried samples were transferred into separate containers, and 2 L of methanol and ethanol were added to each. These solutions were kept dark for 96 h (4 days). After this period, the solutions were filtered through filter paper. Subsequently, the solvents were evaporated using a rotary evaporator (Heidolph, Germany) under reduced pressure at 40 °C and stored at + 4 °C until further use (Aytar 2024).

### Determination of antioxidant properties of flower parts

#### Phytochemical studies

##### Total phenolic content

This study mixed equal volumes of the sample and diluted Folin-Ciocalteu reagent. After incubating at room temperature for 3 min, 1 mL of 2% sodium carbonate solution was added. The mixture was then left to incubate in the dark at room temperature for 1 h, followed by measuring the absorbance at 760 nm using a UV spectrophotometer (Singleton and Rossi 1965). The total phenolic content was expressed as gallic acid equivalent (GAE) in milligrams per gram of dried extract (mg GAE/g extract DW). All measurements were performed in triplicate.

### Total flavonoid content

This method was assessed using the AlCl_3_ method with minor adjustments, following the procedure by (Osuna-Ruiz et al. 2016). Extracts were mixed with distilled water, followed by NaNO_2_ (5%) addition and a standing period. Subsequently, AlCl_3_ (10%) was added, and the solution underwent incubation. NaOH (1 M) was introduced, leaving the solution at room temperature. Absorbance was measured using a UV spectrophotometer, and total flavonoid content was quantified as quercetin equivalent (QE) per gram of dried extract (mg QE/g DW). All measurements were performed in triplicate.

### Determination of radical scavenging activity

The DPPH free radical scavenging activity was determined through the following steps (Braca et al. 2001). DPPH (8 mg) was dissolved in ethanol (100 ml) to obtain a solution with an 80 µg/ml concentration. Stock solutions of plant extracts were prepared at a concentration of 1 mg/ml, and serial dilutions were made from these stock solutions. Each solution (2 ml) was mixed with 2 ml DPPH solution. The mixtures were incubated in the dark at room temperature for 30 min. After the incubation period, the absorbance was measured at 517 nm using a UV spectrophotometer. The DPPH free radical scavenging activity (%) was calculated using the following equation:

$$\:DPPH\:scavenging\:activity\:\left(\%\right)=\frac{Acontrol-Asample}{Acontrol}\times\:100$$

The extract concentration needed to lower the initial DPPH concentration by 50% was determined by building an extract concentration curve against a percentage inhibition scale. The IC50 is the value determined by linear regression analysis. Butylated Hydroxytoluene (BHT) was used as a reference standard. All measurements were performed in triplicate.

### Determination of the chelating ability of ferrous ions

Spectroscopic methods were used to evaluate the binding affinity of Fe²⁺ ions with a reagent. The chelation of Fe²⁺ by Ferrozine was assessed using the method (Dinis et al. 1994). Various quantities of sample and standard compounds were mixed with 0.05 mL of FeCl₂ solution (2 mM). The reaction was initiated by adding 0.2 mL of Ferrozine reagent (5 mM), and the mixture was stirred and left at 25 °C for 10 min. Absorbance values were recorded at 562 nm. Ethylenediaminetetraacetic acid (EDTA) was used as a reference standard. All measurements were performed in triplicate. Using the following formula, the percentage of ferrous ion chelating capacity was determined:

$$\:DPPH\:scavenging\:activity\:\left(\%\right)=\frac{Acontrol-Asample}{Acontrol}\times\:100$$

### Antimicrobial activity

The antimicrobial activity of the plant extract was evaluated against various bacterial and yeast strains, including Gram-positive (e.g., *Staphylococcus aureus* ATCC 25923, *Enterococcus faecalis* ATCC 29212, *Bacillus cereus* NRRL B-3711) and Gram-negative strains (e.g., *Klebsiella pneumoniae* ATCC 13883, *Escherichia coli* ATCC 25922, *Proteus vulgaris* RSKK 96029, *Pseudomonas aeruginosa* ATCC 27853) and yeast (*Candida albicans* ATCC 10231).

For antimicrobial analysis, the extracts were dissolved in DMSO, sterilised through syringe filters with a pore size of 0.45 mm, and prepared at a concentration of 250 mg mL^− 1^.

Cultures were grown in Mueller Hinton Broth and Sabouraud Dextrose Broth at 37 °C and 30 °C, respectively, and adjusted to 0.5 McFarland standard. The broth microdilution method, following CLSI guidelines, was used to determine the extract’s minimal inhibitory concentration (MIC). Ampicillin, chloramphenicol, and ketoconazole were used as positive controls. The minimal bactericidal concentration (MBC) and minimal fungicidal concentration (MFC) were determined by subculturing from non-turbid wells and spotted inoculating onto suitable growth media.

### Gas chromatography-mass spectrometry analysis

The optimisation structures were analysed following the Gas Chromatography-Mass Spectrometry (GC-MS) analysis method by (Aytar 2024). The sample was centrifuged at 3500 rpm for 10 min, and the supernatant was used for GC-MS analysis. The GC-MS analysis was conducted using a SHIMADZU GCMS-QP2010 Mass Spectrometer coupled with an AOC-5000 Auto-Injector. An Rxi-5MS column (30 m x 0.25 mm x 0.25 μm) was used for the analysis, with a scanning range of 30–450 Da. The seed coat samples extracted with methanol using the liquid sampling method were diluted 100 times and placed in 1.5 ml bottles for analysis. The NIST Standard Reference Database was utilised for further analysis and identification of compounds.

### In silico molecular docking analysis

The docking procedure was performed as described by (Biovia 2019). Specifically, the structures of compounds were drawn using ChemDraw 22.0 and saved in (.mol file) format, then optimised using Discovery Studio Visualizer 21.1 software and saved in (.pdb file) format. All target enzymes were downloaded from the protein database and saved in (.pdb file) format. Subsequently, binding sites were identified using Discovery Studio Visualizer software, and all ligands and water molecules were removed from the target proteins. The interaction between ligands and target enzymes was analysed using Discovery Studio Visualizer to show in both 2D and 3D structural formats.

### Prediction of ADMET and topology

Understanding how a substance interacts with the human body involves considering its absorption, distribution, metabolism, excretion, and potential toxicity, collectively known as ADMET features. Evaluating the pharmacokinetic profile, which encompasses these ADMET characteristics, is pivotal for assessing the pharmacodynamic activity of a therapeutic molecule. Nowadays, researchers can benefit from various online and offline software tools, such as the ADMET predictor and SwissADME (https://www.swissadme.ch/), which aid in predicting the behaviour of potential drug candidates. Marvin Sketch 5.0 is valuable for analysing elemental composition, ligand surface characteristics, molecular shape, and other molecular descriptors. By visualising molecular boundaries and interaction surfaces, this tool provides insights into the behaviour of molecules and their interactions with surrounding molecules. These insights hold promise for advancing drug development and short-peptide supramolecular chemistry (Hasan et al. 2022).

### Statistical analysis

All the data were subjected to statistical analysis using IBM SPSS Statistics 22 software.

## Results and discussion

### Phytochemical studies and antioxidant activity

The antioxidant properties and total phenolic and flavonoid contents of ethanol and methanol extracts derived from *B. trixago* flowers were determined (Table 1).

**Table 1:** DPPH radical scavenginactivities and iron chelating B. *Trixago* flower ethanol and methanol extracts (IC_50_ (mg/mL) ± SD) and total phenolic and flavonoid contents ± SD* values

Plant Name	DPPH (IC_50_ mg/mL)	Iron Chelating (IC_50_ mg/mL)	Total Phenolic Compound (mg GAE/g extract DW)	Total Flavonoid Compound (mg QE/g extract DW)
*B.trixago* ethanol	0.95 ± 0.09	8.21 ± 0.09	79.14 ± 2.30	244.31 ± 12.51
*B.trixago* methanol	0.370 ± 0.002	6.68 ± 0.28	51.50 ± 1.43	251.67 ± 6.68
BHT (Positive Control)	0.23 ± 0.01	-	-	-
EDTA (Positive Control)	-	5.30 ± 4.44	-	-

The antioxidant activity of the ethanol extract from *B. trixago* flowers was found to be 0.95 ± 0.09 mg/mL. The iron chelation capacity of the ethanol extract from *B. trixago* flowers was measured at 8.21 ± 0.09 mg/mL. The total phenolic content was 79.14 ± 2.30 mg GAE/g extract DW, while the total flavonoid content was 244.31 ± 12.51 mg QE/g extract DW.

The antioxidant activity of the methanol extract from *B. trixago* flowers was higher than that of the ethanol extract, measuring 0.370 ± 0.002 mg/mL. The iron chelation capacity of the methanol extract from *B. trixago* flowers was determined to be 6.68 ± 0.28 mg/mL, and this activity was found to be better than that of the ethanol extract. The total phenolic content was measured as 51.50 ± 1.43 mg GAE/g extract DW, while the total flavonoid content was 251.67 ± 6.68 mg QE/g extract DW.

The IC_50_ values for DPPH radical scavenging activity of the positive controls BHT and EDTA were determined to be 0.23 ± 0.01 mg/mL for DPPH radical scavenging activity and 5.30 ± 4.44 mg/mL for iron chelation, respectively. These results emphasise the good antioxidant potential of the methanol extract from *B. trixago* compared to the positive controls.

Additionally, it was determined that the total phenolic content of the ethanol extract from *B. trixago* flowers was higher than the methanol extract. In contrast, the total flavonoid content was better in the methanol extract.

The total phenolic content of *E. officinalis* aerial parts, extracted with 70% ethanol, was found to be 92.10 ± 2.90 mg GAE/g, while the total flavonoid content was 24.72 ± 0.29 mg RE/g. The DPPH antioxidant activity showed an IC_50_ value of 50.93 ± 2.19 µg/mL. In comparison, *E. stricta* demonstrated a total phenolic content of 74.91 ± 1.28 mg GAE/g and a total flavonoid content of 10.81 ± 0.19 mg RE/g, with a DPPH IC_50_ value of 71.57 ± 3.42 µg/mL. The total phenolic content of *E. officinalis* aerial parts, extracted with 70% ethanol, is higher compared to the ethanol extract of *B. trixago* flowers, but its flavonoid content is lower. The total phenolic content of *E. stricta* aerial parts, obtained with 70% ethanol, is nearly the same as that of *B. trixago* flowers, and its flavonoid content is lower. However, the antioxidant activity in the *B. trixago* ethanol extract is much lower compared to the other two extracts. The total phenolic content of *E. officinalis* aerial parts, extracted with 70% ethanol, is higher than the methanol extract of *B. trixago* flowers, but its flavonoid content is lower. The total phenolic content of *E. stricta* aerial parts, obtained with 70% ethanol, is higher than that of *B. trixago* flowers, while its flavonoid content is lower. However, the antioxidant activity in the *B. trixago* methanol extract is much lower compared to the other two extracts (Benedec et al. 2024).

The ethanolic extract of *Cistanche violacea* exhibited a total phenolic content of 183.51 ± 2.2 µg GAE/mg DE and a total flavonoid content of 40.26 ± 6.02 µg QE/mg DE. The aqueous extract, on the other hand, showed a total phenolic content of 79.48 ± 2.4 µg GAE/mg DE and a total flavonoid content of 17.05 ± 0.1 µg QE/mg DE. Additionally, it has been reported that the IC_50_ value of the DPPH test for the ethanolic extract obtained via maceration of *C. violacea* is 33.35 ± 1.4 µg/mL. In contrast, the aqueous extract has an IC_50_ value of 78.73 ± 1.4 µg/mL. Compared to the ethanolic extract obtained from *C. violacea* through maceration, the ethanolic and methanolic extracts from *B. trixago* flowers exhibited lower antioxidant activity despite having high phytochemical contents (Djemouaı et al. 2024).

DPPH radical scavenging activity of *Centranthera grandiflora* root extract has an IC_50_ value of 0.166 mg/mL. The antioxidant activities of the ethanol and methanol extracts from *B. trixago* flowers were lower than the *C. grandiflora* root extract, although both extracts demonstrated good antioxidant activity (Xin-lan et al. 2024).

According to the experimental analysis results, the aqueous extracts obtained from the roots of *Cistanche phelypaea* exhibited total phenolic contents of 92.45 ± 0.73 mg GAE/g extract using the decoction method, 79.12 ± 1.27 mg GAE/g extract using the infusion method, and 78.59 ± 4.56 mg GAE/g extract using the cold maceration method. The total flavonoid content was determined to be 14.26 ± 0.54 mg QE/g extract using the decoction method, 14.71 ± 0.30 mg QE/g extract using the infusion method, and 9.85 ± 0.55 mg QE/g extract using the cold maceration method. Regarding antioxidant activity, the IC₅₀ values were found to be 19.545 ± 0.993 µg/mL for the decoction method, 19.061 ± 0.211 µg/mL for the infusion method, and 22.748 ± 1.498 µg/mL for the cold maceration method. In our study, the total phenolic content of the ethanol extract from B. trixago flowers was lower than that of the decoction method of *C. phelypaea* roots. Still, it was similar to the infusion and cold maceration methods. Additionally, the flavonoid content of the *B. trixago* flowers’ ethanol extract was approximately 17 to 27 times higher than that of the *C. phelypaea* roots across all three extraction methods. The total phenolic content of the *B. trixago* flowers’ methanol extract was lower than that of the ethanol extract. The flavonoid content was still significantly higher across all extraction methods. Finally, the antioxidant activity of the ethanol and methanol extracts of B. trixago flowers was considerably lower than the three extraction methods of *C. phelypaea* roots (Rahim et al. 2022),.

The IC_50_ values for the extracts of *Orobanche caryophyllacea*, *Phelipanche arenaria*, and *Phelipanche ramosa* in the DPPH assay, which were found to be 275.94 ± 0.69 µg/mL, 311.40 ± 1.93 µg/mL, and 318.63 ± 0.32 µg/mL, respectively. The ethanol and methanol extracts obtained from *B. trixago* flowers had higher IC_50_ values than the antioxidant activities of *O. caryophyllacea*,* P. arenaria*, and *P. ramosa* (Skalski et al. 2021). Consequently, the antioxidant activity of the methanol extract from *B. trixago* flowers is lower than those of *O. caryophyllacea*,* P. arenaria*, and *P. ramose.*

The phenolic content of the methanolic extract of *Orobanche crenata* was quantified as 762.5 ± 11.5 mg GAE/g extract DW. In our study, we observed that the total phenolic contents of the ethanol and methanol extracts obtained from *B. trixago* flowers were lower than the phenolic content of the methanol extract of *O. crenata* (Hegazy et al. 2020).

Methanol extracts of *O. crenata* exhibited a total phenolic content of 19.99 mg EAG/g extract DW, which was notably higher than the 3.42 mg EAG/g extract DW observed in the methanol extracts of *Orobanche foetida*. Additionally, the total phenolic content of *O. crenata* water extracts was determined to be 3.02 mg EAG /g extract DW, whereas *O. foetida* water extracts exhibited a total phenolic content of 4.24 mg EAG/g extract DW. The *O. foetida* methanol extract demonstrated an inhibition rate of 91.97% in DPPH radical scavenging tests, while *O. crenata* exhibited an effective inhibition rate of 88.11%. Both extracts showed strong antioxidant activities compared to standard substances such as BHT and ascorbic (Abbes et al. 2014). Our study observed higher phenolic content of the ethanol and methanol extracts obtained from *B. trixago* flowers. Abbes et al. reported a high inhibitory effect of the methanol extracts of *O. foetida* and *O. crenata* in the DPPH radical scavenging test (Abbes et al. 2014). In contrast, our study found that the ethanol and methanol extracts obtained from *B. trixago* flowers exhibited good antioxidant activity.

In the study by (Nikolova et al. 2014), the DPPH radical scavenging activity of *Bartsia alpina* (= *Bellardia alpina*) was determined with an IC_50_ value of 116.6 µg/mL and the total phenolic content was found to be 104.94 ± 9.91 mg GAE/g extract DW. Our study determined that the total phenolic contents of the ethanol and methanol extracts obtained from *B. trixago* flowers were lower than the phenolic content of the methanol extract of *B. alpina* reported by Nikolova et al. Furthermore, it was also found that the antioxidant activities of these extracts were low.

In the literature, studies currently need to be available addressing the phenolic content activity, antioxidant activity, and iron chelation activity related to *B. trixago*. A literature review on *B. alpina* (formerly known as *B. alpina*) revealed only one available study. However, *B. trixago* has demonstrated strong antimicrobial and antioxidant activities compared to other species within its family. This indicates that the plant could be a significant source of potential health benefits. Mainly, phenolic compounds are crucial factors in determining the positive effects of plants on health, and research in this area can enhance our understanding of the characteristics and potential advantages of these plants. The activities exhibited by *B. trixago* warrant further investigation, as this could yield valuable insights for both the pharmaceutical and food industries. Thus, exploring these plants and evaluating their phenolic content represents an important area for future research.

### Antimicrobial activity of *B. trixago* flower ethanol and methanol extracts against various microorganisms

In this study, the antimicrobial activities of *B. trixago* flower ethanol and methanol extracts against various microorganisms were compared with standard drugs such as Ampicillin (Amp), Chloramphenicol (C), and Ketoconazole (Keto). MIC and MBC values of B. trixago flower ethanol and methanol extracts against the tested bacteria are presented in Table 2. According to the results obtained, MIC and MBC values ranged from < 0.39 mg/mL to > 125 mg/mL. Based on the antimicrobial screening results, the MIC values of *B. trixago* flower ethanol extract ranged from 31.25 mg/mL to > 125 mg/mL for Amp, from 15.63 mg/mL to > 125 mg/mL for C and 31.25 mg/mL for Keto. Similarly, the MIC values of *B. trixago* flower methanol extract ranged from 31.25 mg/mL to > 125 mg/mL for Amp, from 15.63 mg/mL to > 125 mg/mL for C and 31.25 mg/mL for Keto. All extracts exhibited strong bactericidal and bacteriostatic effects against the tested bacteria. The highest antimicrobial activity was observed with *B. trixago* ethanol and methanol extracts against the *B. cereus* NRRL B-3711 strain. Furthermore, the *B. trixago* ethanol extract demonstrated the second-highest significant antimicrobial activity against the *K. pneumoniae* strain compared to the methanol extract.

**Table 2:** Antimicrobial activity of *B.trixago*

				Positive Control	Positive Control	Positive Control
Microorganisms		*B. trixago flowers* ethanol (mg/mL)	*B. trixago* flowers methanol(mg/mL)	Amp(mg/mL)	C(mg/mL)	Keto(mg/mL)
*E. coli* ATCC 25,922	MIC	6.25	12.5	> 125	> 125	NS
	MBC	6.25	12.5	> 125	125	NS
*S. aureus* ATCC 25,923	MIC	6.25	6.25	> 125	> 125	NS
	MBC	12.5	12.5	62.5	125	NS
*K. pneumoniae* ATCC 13,883	MIC	3.13	6.25	62.5	> 125	NS
	MBC	3.13	6.25	125	15.63	NS
*P. vulgaris* RSKK 96,029	MIC	6.25	12.5	125	15.63	NS
	MBC	6.25	50	> 125	125	NS
*E. faecalis* ATCC 29,212	MIC	6.25	12.5	> 125	> 125	NS
	MBC	50	25	> 125	> 125	NS
*B. cereus* NRRL B-3711	MIC	< 0.39	< 0.39	31.25	125	NS
	MBC	< 0.39	< 0.39	> 125	125	NS
*P. aeruginosa* ATCC 27,853	MIC	6.25	12.5	> 125	125	NS
	MBC	6.25	12.5	> 125	> 125	NS
*C. albicans* ATCC 10,231	MIC	6.25	6.25	NS	NS	31.25
	MFC	12.5	12.5	NS	NS	62.5

(Amp: Ampicillin; C: Chloramphenicol; Keto: Ketoconazole; NS: Not Studied)

According to the results of (Djemouaı et al. 2024), the ethanol extract of *C. violacea* obtained by the maceration method showed no inhibition against bacterial strains ranging from *E. coli* to *B. cereus*. The inhibition zone for both extracts was recorded as 6 mm, whereas the positive control exhibited a broader inhibition zone. This indicates that the *C. violacea* extracts do not possess effective antimicrobial activity. Ethanol and methanol extracts obtained from *B. trixago* flowers exhibited promising antimicrobial activity against *E. coli* and *S. aureus* bacterial strains compared to the positive control; however, *C. violacea* showed no antimicrobial activity compared to these extracts.

According to the antimicrobial analysis results (Benedec et al. 2024), the *E. officinalis* aerial parts extracted with 70% ethanol showed inhibition zones of 13.75 ± 0.43 mm for *B. cereus*, 9.75 ± 0.43 mm for *E. coli*, and 10 ± 0.00 mm for *C. albicans*. Additionally, no activity was observed against *P. aeruginosa*. The *E. stricta* aerial parts extracted with 70% ethanol exhibited inhibition zones of 15.00 ± 0.00 mm *for B. cereus*, 9.75 ± 0.43 mm for *E. coli*, and 10 ± 0.00 mm for *C. albicans*. No activity was observed against *P. aeruginosa* or *C. albicans.* The ethanol and methanol extracts of *B. trixago* flowers show antimicrobial activity against *C. albicans*, compared to the 70% ethanol extract of *E. stricta* aerial parts. However, against *P. aeruginosa*, no activity was observed in the 70% ethanol extracts of *E. officinalis* and *E. stricta* aerial parts, whereas *B. trixago* flowers ethanol and methanol extracts exhibited good activity.

In a study conducted by (Bouzayani et al. 2022), it was demonstrated that the methanol extracts of *Cistanche violacea* exhibited significant antibacterial activity against both Gram-positive and Gram-negative strains, with inhibition zones ranging from 10 to 18.6 mm for Gram-positive strains and from 9 to 18.3 mm for Gram-negative strains. The ethanol extracts of *C. violacea* displayed varying antibacterial activity, ranging from 7.3 to 11.6 mm, while the n-hexane and acetone-water extracts showed no antibacterial activity. In our study, it was observed that both the ethanol and methanol extracts of *B. trixago* demonstrated strong antibacterial activity against Gram-positive bacterial strains, particularly *S. aureus*,* E. faecalis*, and *B. cereu*s, as well as Gram-negative strains, including *E. coli*,* K. pneumoniae*,* P. vulgaris*, and *P. aeruginosa.*

In the study by (Jaradat et al. 2021), the methylene chloride extract of *Orobanche aegyptiaca* demonstrated the highest antibacterial activity against *S. aureus*, with a reported MIC value of 0.78 mg/ml. Additionally, the methylene chloride, petroleum ether, and chloroform extracts of *O. aegyptiaca* exhibited potential antifungal activity against *C. albicans* strains, with MIC values established at 0.78 mg/ml. Our study observed that the ethanol extract of *B. trixago* exhibited similarly strong antibacterial activity against *S. aureus* bacteria and *Candida* fungal strains compared to the methanol extract. This result indicates that the ethanol extract of *B. trixago* could be evaluated as a potential antifungal and antibacterial agent.

In a study by (Musa et al. 2021), the volatile components of *Cistanche tubulosa* obtained through hydro distillation exhibited inhibitory activity of 2.23 mg/100 mL against *S. aureus* and 15.68 mg/100 mL against *B. cereus*. Additionally, inhibitory activity was observed at 18.35 mg/100 mL for *E. coli* and 31.61 mg/100 mL for *K. pneumoniae.* Promising antifungal activity was also reported against *C. albicans*, with an inhibitory value of 4.36 mg/mL. Compared to the volatile components of *C. tubulosa*, the *B. trixago* flowers and methanol-ethanol extract exhibited lower antimicrobial activity against *S. aureus* bacteria but much higher activity against *B. cereus* bacteria. The ethanol and methanol extracts also demonstrated high antimicrobial activity against *E. coli* and *K. pneumoniae* bacteria. However, lower activity was observed against the *C. albicans* fungal suspension.

In a comprehensive assessment by (Genovese et al. 2020), it was reported that the acetonic extract derived from the leaves of *O. crenata* exhibited MIC values ranging from 94.10 to 376.00 µg/ml against *C. albicans* strains and from 94.10 to 3011.00 µg/ml against non-albicans *Candida* strains. These findings underscore the fungicidal potential of the acetonic extract. Our study observed that the ethanol extract of *B. trixago* exhibited similarly strong antibacterial activity against *Candida* fungal strains compared to the methanol extract. This result suggests that the ethanol extract of *B. trixago* could be evaluated as a potential antifungal agent.

The methanol extract of *O. foetida e*xhibited activity against all bacterial strains except for *S. aureus*, forming clear inhibition zones ranging from 12 to 30 mm. In contrast, the methanol extract of *O. crenata* inhibited only *Listeria monocytogenes* and *Salmonella enteritidis*, with inhibition zones measuring 10 mm and 25 mm, respectively. These findings indicate that the methanol extracts showed higher activity compared to the aqueous extracts. Furthermore, it was determined that the aqueous extracts from both *Orobanche* species exhibited no activity against the bacterial isolates (Abbes et al. 2014). Our study observed that the ethanol extract of *B. trixago* exhibited similarly strong antibacterial activity against *S. aureus* compared to the methanol extract. Both the ethanol and methanol extracts of *B. trixago* were effective against bacterial strains such as *E. coli*,* K. pneumoniae*,* P. vulgaris*,* E. faecalis*,* P. aeruginosa*, and *B. cereus*, as well as showing antibacterial activity against *Candida* fungal strains. Notably, the ethanol extract exhibited a stronger antibacterial effect against the other bacteria except for the *S. aureus* strain; both extracts demonstrated similarly strong antimicrobial effects against the *Candida* fungal strains.

The ethanol extracts of *O. crenata* exhibited moderate antibacterial activity against three Gram-positive bacteria (*S. aureus*,* B. subtilis*, and *S. faecalis*) and three Gram-negative bacterial strains (*E. coli*, *P. aeruginosa*, and *N. gonorrhoeae*) (Nada and El-Chaghaby 2015). Our study observed that the ethanol extract of *B. trixago* exhibited stronger activity against *E. coli*, *P. aeruginosa*, *K. pneumoniae*, *P. vulgaris* (Gram-negative bacteria) and *E. faecalis* (Gram-positive bacteria) compared to the methanol extract. *K. pneumoniae* demonstrated the highest antimicrobial activity against the other strains. Furthermore, it was determined that the ethanol and methanol extracts of *B. trixago* showed similarly strong antimicrobial activities against *B. cereus* and *S. aureus* (Gram-positive bacteria). Notably, *B. cereus* exhibited a stronger antimicrobial activity compared to *S. aureus*. The ethanol and methanol extracts of *B. trixago* also demonstrated antifungal activity against *C. albicans* fungal strains. These findings indicate that the ethanol extract of *B. trixago* presents a broader antibacterial spectrum and stronger antibacterial activity, thus possessing higher potential compared to the ethanol extract of *O. crenata*. The high activity of *B. trixago* against various bacterial species supports its consideration as a potential antibacterial agent. On the other hand, the moderate activity exhibited by the ethanol extract of *O. crenata* suggests that the antibacterial activity of this plant is limited.

In the different study, the antibacterial activity of ethanol extracts derived from the shoots *of O. crenata* against the plant pathogen *Erwinia* was evaluated, revealing that the extract inhibited bacterial growth at a concentration of 63,000 µg/ml, resulting in an inhibition zone diameter of 10–12 mm (Saadoun et al. 2008). Our study found that the ethanol extract of *B. trixago* exhibited a more potent antibacterial effect against various bacterial strains than the methanol extract. The ethanol and methanol extracts of *B. trixago* demonstrated the strongest antimicrobial activity against *B. cereus*. Furthermore, the ethanol extract of *B. trixago* showed potent activity against *K. pneumoniae*, following *B. cereus*.

### Gas chromatography-mass spectrometry analysis results of methanol and ethanol extracts of *B. trixago* flowers

A total of 25 compounds were identified in the methanol extract of *B. trixago* flowers, while 20 compounds were detected in the ethanol extract, as shown in Tables 3 and 4. Among the compounds with major peak areas in the methanol extract, stigmasterol stood out with a percentage of 12.65%, l-(+)-Ascorbic acid 2,6-dihexadecanoate with 5.86%, and 9,12,15-Octadecatrienoic acid, (Z, Z,Z)- with 6.47%. Additionally, Hexadecanoic acid, 2-hydroxy-1-(hydroxymethyl)ethyl ester had a peak area percentage of 2.99%, Azulene, 1,2,3,4,5,6,7,8-octahydro-1,4-dimethyl-7-(1-methylethenyl)-, (1 S,4 S,7R)- with 2.15%, 7-(Iodomethyl)-3a-isopropenyl-6,7-dimethyloctahydro-1 H-indene with 3.83%, and 2,7-Dioxa-tricyclo[4.4.0.0(3,8)]deca-4,9-diene with 2.97%.

**Table 3:** GC-MS analysis results of *B. trixago flowers* ethanol extract

No	Retention Time (minutes)	Compound Name	Molecular Formula	Molecular Weight	Area (%)
1	3.213	Dihydroxyacetone	C_3_H_6_O_3_	90.08	4.77
2	6.159	Oxirane, [(hexyloxy)methyl]-	C_6_H_12_O_2_	116.15	0.56
3	7.150	Glycerin	C_3_H_8_O_3_	92.09	0.48
4	8.718	Benzofuran	C_8_H_6_O	118.13	0.61
5	11.726	1,3,5-Triazine-2,4,6-triamine	C_3_H_6_N_6_	126.11	0.79
6	13.911	4 H-Pyran-4-one, 2,3-dihydro-3,5-dihydroxy-6-methyl-	C_6_H_8_O_4_	144.12	1.18
7	17.253	2,7-Dioxa-tricyclo[4.4.0.0(3,8)]deca-4,9-diene	C_8_H_8_O_2_	136.15	2.97
8	18.077	Benzoic acid	C_7_H_6_O_2_	122.12	4.76
9	20.252	2-Nonen-4-yne, (E)-	C_9_H_14_	122.21	1.17
10	23.018	4-Hydroxy-2-methylacetophenone	C_9_H_10_O_2_	150.17	0.51
11	24.470	Tricyclo[4.4.0.0(2,8)]decan-9-ol	C_10_H_16_O	152.23	0.47
12	27.238	3,6-Nonadien-1-ol	C_9_H_16_O	140.22	0.44
13	37.982	4,7-Epoxyisobenzofuran-1,3-dione, hexahydro-3a,7a-dimethyl-, (3aalpha,4beta,7beta,7aalpha)-	C_10_H_12_O_4_	196.20	1.11
14	43.879	Tetradecanoic acid	C_14_H_28_O_2_	228.37	0.85
15	48.159	l-(+)-Ascorbic acid 2,6-dihexadecanoate	C_38_H_68_O_8_	652.90	5.86
16	48.697	Azulene, 1,2,3,4,5,6,7,8-octahydro-1,4-dimethyl-7-(1-methylethenyl)-, (1 S,4 S,7R)-	C_15_H_24_	204.35	2.15
17	46.740	7-Oxocholesteryl acetate	C_24_H_50_	338.70	1.08
18	50.439	9,12,15-Octadecatrienoic acid, (Z, Z,Z)-	C_18_H_30_O_2_	278.42	6.47
19	50.632	Octadecanoic acid	C_18_H_36_O_2_	284.47	1.72
20	51.977	Heneicosane	C_21_H_44_	296.60	0.96
21	52.138	7-(Iodomethyl)-3a-isopropenyl-6,7-dimethyloctahydro-1 H-indene	C_15_H_25_I	332.26	3.83
22	52.593	Stigmasterol	C_20_H_34_O	290.50	12.65
23	52.797	alpha-Terpinene	C_10_H_16_	136.23	1.99
24	53.351	3-Methyl-4-(1,3,3-trimethyl-7-oxa-bicyclo[4.1.0]hept-2-yl)-but-3-en-2-one	C_14_H_22_O_2_	222.32	1.57
25	54.62	Hexadecanoic acid, 2-hydroxy-1-(hydroxymethyl)ethyl ester	C_19_H_38_O_4_	330.50	2.99

In the ethanol extract, when examining the compounds with major peak areas, stigmasterol stood out with a percentage of 26.51%, l-(+)-Ascorbic acid 2,6-dihexadecanoate with 5.01%, and 7-(Iodomethyl)-3a-isopropenyl-6,7-dimethyloctahydro-1 H-indene with 5.59%. Additionally, Hexadecanoic acid, 2-hydroxy-1-(hydroxymethyl) ethyl ester had a peak area percentage of 3.50%, Tetracosamethyl-cyclododecasiloxane with 2.76%, 1-Dodecanol with 1.30%, Anthracene, 9-dodecyltetradecahydro- with 1.06%, Cycloheptane, 1-ethenyl-1-methyl-4-methylene-2-(2-methyl-1-propenyl)- with 1.84%, and Hexatriacontane with 1.65%.

In our study, GC-MS analysis identified stigmasterol in the ethanol and methanol extracts of *B. trixago*. Stigmasterol has demonstrated anti-inflammatory (Jie et al. 2022), anti-diabetic (Zhang et al. 2022), immunomodulatory, antiparasitic, antifungal, antibacterial, antioxidant, and neuroprotective properties (Bakrim et al. 2022). Notably, stigmasterol can perform neuroprotective functions in central nervous system disorders like Alzheimer’s disease, multiple sclerosis, and amyotrophic lateral sclerosis/parkinsonism dementia (Haque et al. 2021). Its ability to cross the blood-brain barrier makes it a unique compound (Suping et al. 2023). Moreover, stigmasterol has demonstrated anti-tumour potential against various types of cancer, such as lung (Song et al. 2022), gallbladder (Pandey et al. 2019), gastric (Zhao et al. 2021), and ovarian cancers (Bae et al. 2020), both in vitro and in vivo. The mechanisms underlying its anti-cancer effects include increasing ROS production, raising Ca^2+^ levels in the cytosol and mitochondria, inducing mitochondrial depolarisation, promoting apoptosis, suppressing cell migration, and inhibiting the activity of angiogenesis genes in tumour cells (Bakrim et al. 2022). Interestingly, its neuroprotective effects have been linked to its ability to reduce oxidative stress, lower ROS and lipid peroxidation levels, and inhibit apoptosis (Pratiwi et al. 2021). However, the molecular mechanisms underlying its neuroprotective effects remain incompletely understood. Stigmasterol, a precursor for progesterone synthesis, also serves as an intermediate in synthesising estrogens, androgens, corticoids, and vitamin D3. It was identified in the aerial parts of *Viola odorata* through hexane extract using GC-MS analysis (Ali et al. 2024). Furthermore, the leaf extract of *Pseuderanthemum palatiferum* was shown to contain stigmasterol, which exhibits anti-diabetic activity by enhancing the translocation of glucose transporter type 4 (GLUT4), improving insulin resistance, reducing fasting glucose levels, and inducing β-cell regeneration (Alawode et al. 2021). Another study reported that stigmasterol identified in *Aloe perryi* petroleum ether extract activates pro-apoptotic proteins and enhances antiproliferative inhibitory activity in breast cancer (Farshori et al. 2022). Additionally, stigmasterol identified in the ethanolic extract of *Annona muricata* leaves exhibited anticancer activity against HeLa and Vero cells (Ahamed et al. 2022).

GC-MS analysis of the ethanol and methanol extracts of *B. trixago* identified l-(+)-Ascorbic acid 2,6-dihexadecanoate, an ester of ascorbic acid and stearic acid. Ascorbic acid, commonly known as vitamin C, is a potent antioxidant essential for immune system function, skin health, and tissue repair. Stearic acid, a saturated fatty acid, has been shown to exhibit antimicrobial, antioxidant, and anticancer activities (Babouongolo et al. 2021). *B. trixago* methanol extract analysis using GC-MS revealed the presence of 9,12,15-Octadecatrienoic acid, which has been reported to exhibit anticancer properties (Asghar et al. 2011; Hema et al. 2011).

The GC-MS analysis of *B. trixago* extracts identified cembrene as the primary compound, accounting for 51.7%, while the other most abundant components were phellandral at 15.4% and α-terpineol at 14.5% (Semiz and Günal 2023). In another study seco-labdane diterpene alcohol trixagol and its hemi-malonate were the major constituents in *B. trixago* (Morimoto 2019). Due to their viscosities, these compounds may influence insect feeding behaviour and act as a physical defence mechanism against herbivorous insects. Additionally, in a different study *B. trixago* methanol extracts revealed that the compounds 3,5,6,7,8,3’,4’-Heptamethoxyflavone and 3,5,6,7,8,4’-Hexamethoxyflavone exhibited antifungal activity against *Cladosporium herbarum* fungus (Formisano et al. 2008).

### Molecular docking analysis of stigmasterol against selected protein targets

Stigmasterol, a plant sterol of interest due to its potential bioactive properties, has been investigated through docking studies against three protein targets: the Rieske head domain in Complex III2 from *C. albicans* (7RJD), the *E. coli* toxin-antitoxin system HipBST (7AB4), and the large ribosomal subunit of *S. aureus* (4WCE) (Table 5). Docking simulations revealed favourable binding affinities and interaction patterns.

**Table 4:** GC-MS analysis results of *B. trixago* flowers ethanol extract

No	Retention Time (minutes)	Compound Name	Molecular Formula	Molecular Weight	Area (%)
1	5.647	2-Hydroxy-gamma-butyrolactone	C_4_H_6_O_2_	86.09	0.88
2	8.680	Benzofuran	C_8_H_6_O	118.13	0.46
3	16.531	1-Dodecanol	C_12_H_26_O	186.33	1.30
4	17.067	Benzoic acid	C_7_H_6_O_2_	122.12	2.68
5	20.319	3,6-Nonadien-1-ol	C_9_H_16_O	140.22	0.49
6	20.183	4,7-Epoxyisobenzofuran-1,3-dione, hexahydro-3a,7a-dimethyl-, (3aalpha,4beta,7beta,7aalpha)-	C_10_H_12_O_4_	196.20	0.50
7	37.682	2-Isopropyl-5-methyl-6-oxabicyclo[3.1.0]hexane-1-carboxaldehyde	C_10_H_16_O_2_	168.23	0.93
8	48.114	l-(+)-Ascorbic acid 2,6-dihexadecanoate	C_38_H_68_O_8_	652.90	5.01
9	48.686	Cycloheptane, 1-ethenyl-1-methyl-4-methylene-2-(2-methyl-1-propenyl)-	C_15_H_24_	204.35	1.84
10	48.686	7-Oxocholesteryl acetate	C_24_H_50_	338.70	0.65
11	51.878	Anthracene, 9-dodecyltetradecahydro-	C_26_H_48_	360.70	1.06
12	51.977	Tetracosane	C_24_H_50_	338.70	1.21
13	52.135	7-(Iodomethyl)-3a-isopropenyl-6,7-dimethyloctahydro-1 H-indene	C_15_H_25_I	332.26	5.59
14	52.590	Stigmasterol	C_20_H_34_O	290.50	26.51
15	52.791	Andrographolide	C_20_H_30_O_5_	136.23	1.80
16	54.181	alpha-Terpinene	C_10_H_16_	136.23	1.16
17	54.507	Hexatriacontane	C_36_H_74_	507.00	1.65
18	54.632	Hexadecanoic acid, 2-hydroxy-1-(hydroxymethyl)ethyl ester	C_19_H_38_O_4_	330.50	3.50
19	55.775	Lupeol	C_30_H_50_O	426.70	0.37
20	55.955	Tetracosamethyl-cyclododecasiloxane	C_24_H_72_O_12_Si_12_	889.80	2.76

**Fig. 3:**
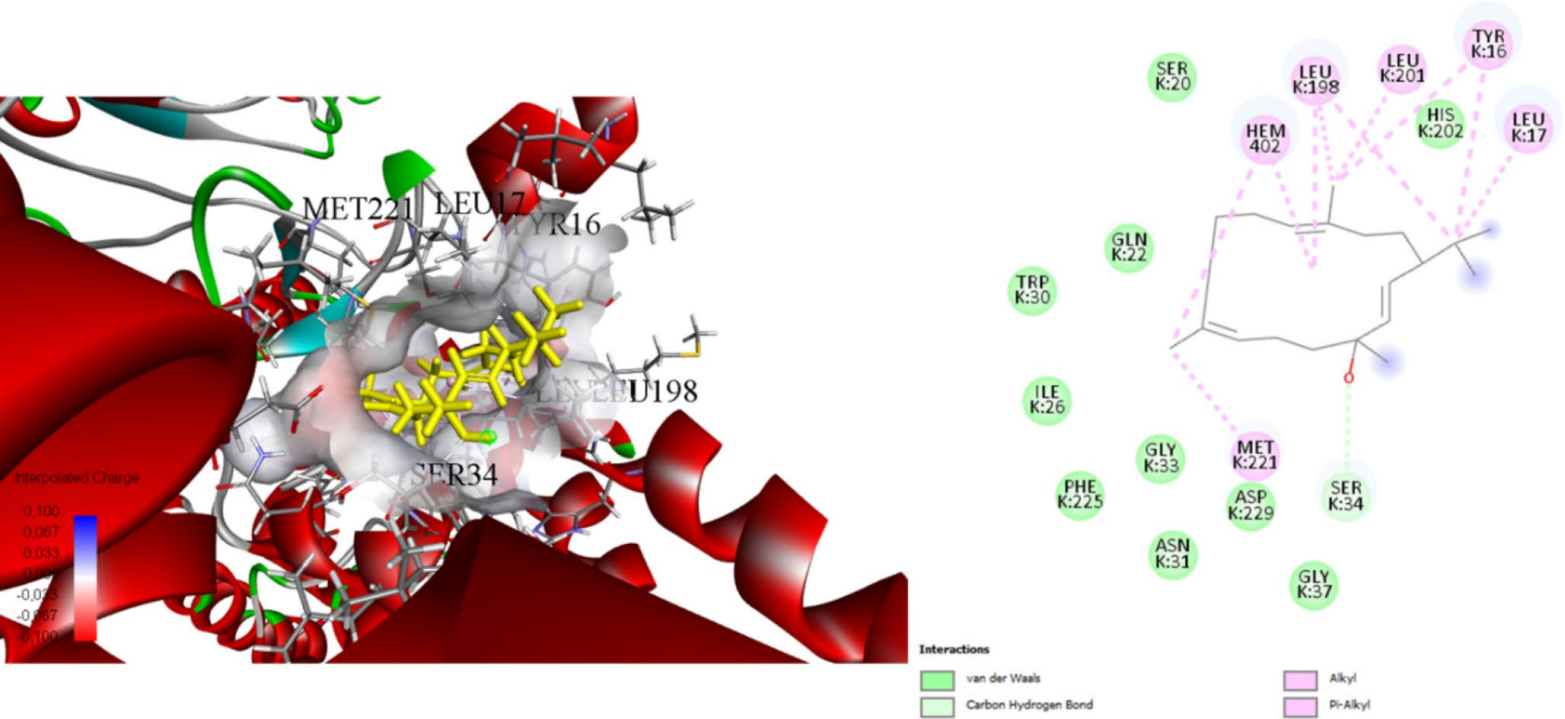
Molecular docking process Stigmasterol with Complex III2 from *Candida albicans*, inhibitor free, Rieske head domain in c position (7RJD)

**Fig. 4:**
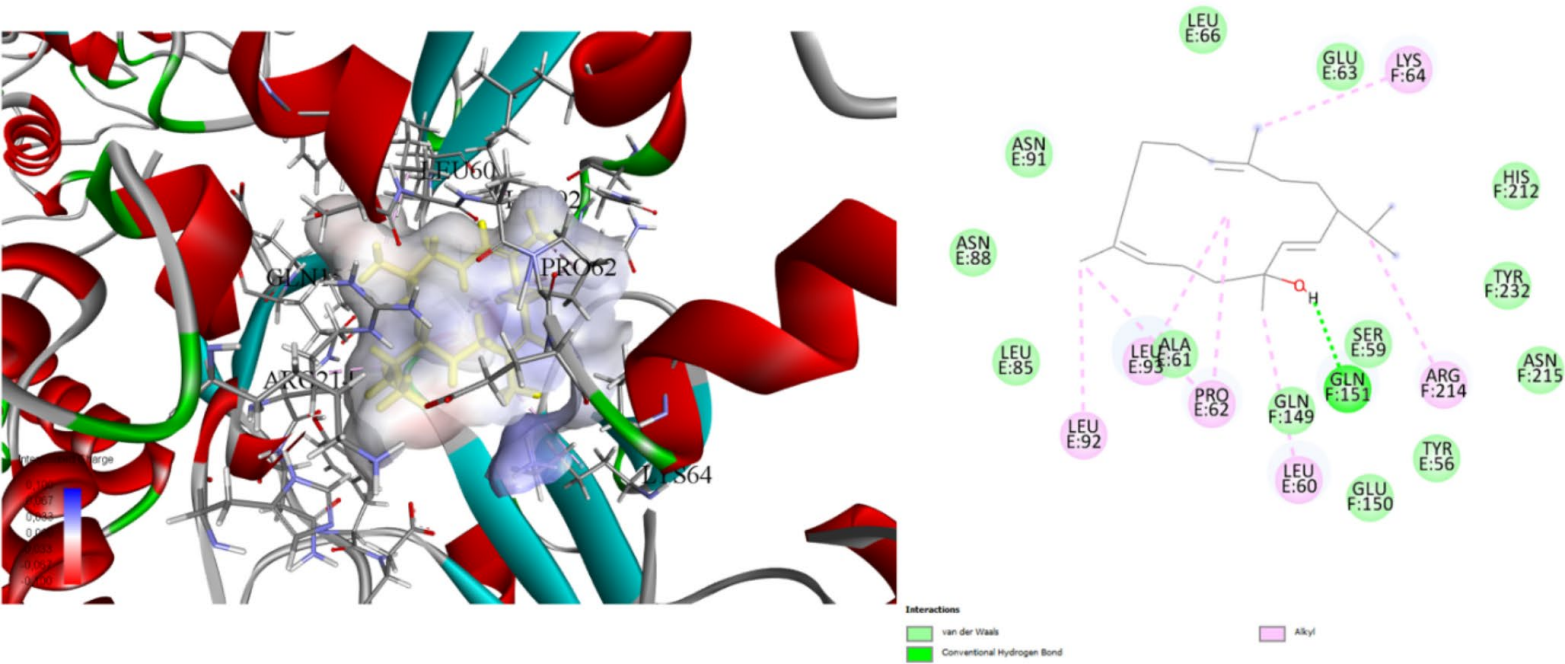
Molecular docking process Stigmasterol with *E. coli* toxin-antitoxin system HipBST (7AB4)

**Fig. 5:**
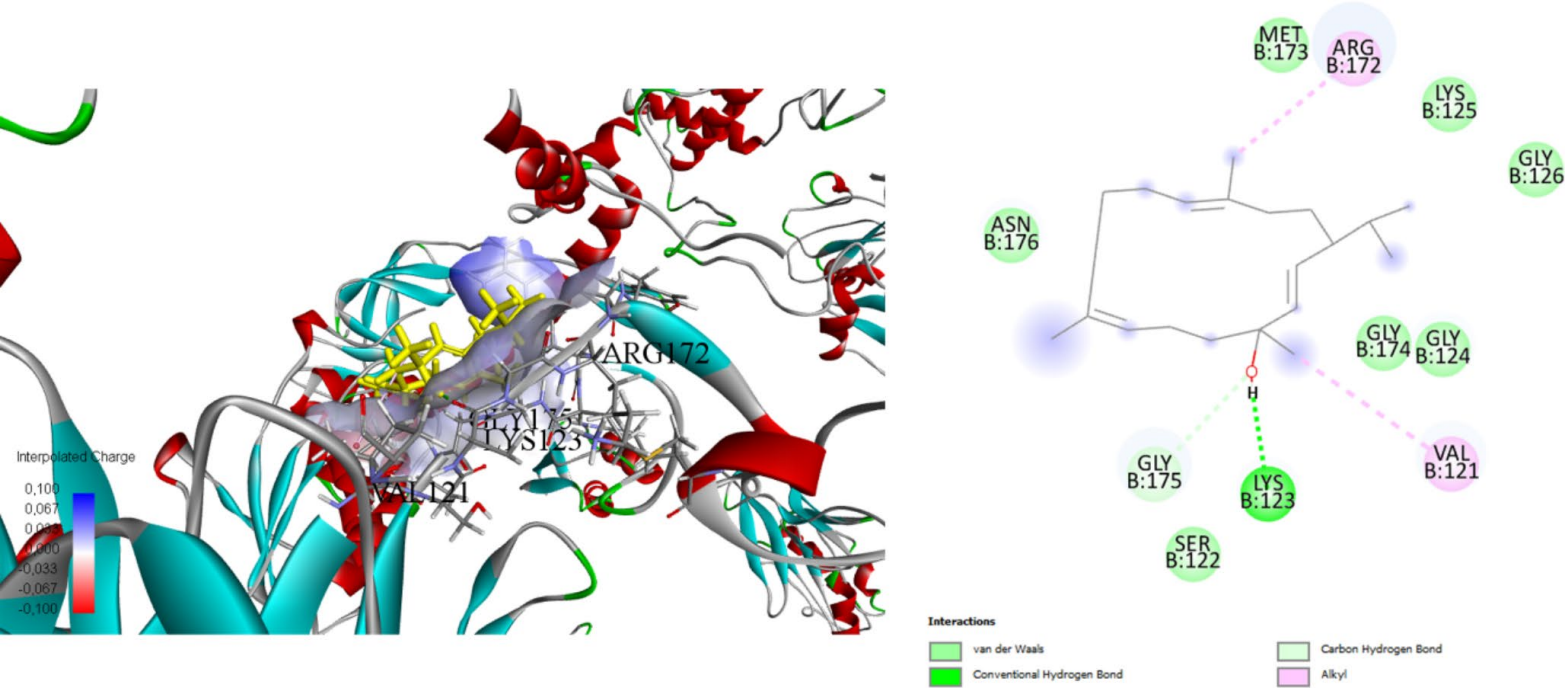
Molecular docking process Stigmasterol with the large ribosomal subunit of *Staphylococcus aureus* (4WCE)

In our study with Complex 7RJD, stigmasterol demonstrated a strong binding affinity with a binding energy of -8.7 kcal/mol. It engaged in alkyl interactions with critical residues such as SER34, LEU198, and MET221, forming carbon-hydrogen bonds ranging from 2.40 to 5.33 Å (Fig. 3). Against HipBST (7AB4), stigmasterol exhibited a binding energy of -7.2 kcal/mol. It formed conventional hydrogen bonds with GLN151 at 2.01 Å and engaged in alkyl interactions with residues PRO62, LEU93, and ARG214, with interaction distances ranging from 3.98 to 5.29 Å (Fig. 4). Docking with the large ribosomal subunit of *S. aureus* (4WCE) resulted in a binding energy of -7.0 kcal/mol. Stigmasterol formed conventional hydrogen bonds with LYS123 (distance: 1.73 Å) and GLY175 (distance: 2.61 Å). Additionally, it engaged in alkyl interactions with VAL121 and ARG172, with interaction distances ranging from 3.78 to 5.05 Å (Fig. 5).

Molecular docking studies show that stigmasterol isolated from *Garcinia wightii* exhibits a strong binding affinity of -9.52 kcal/mol for the 4FDO protein (*Mycobacterium tuberculosis* DprE1 in complex with CT319) (Aswathy et al. 2025). This suggests that stigmasterol could potentially be a promising antitubercular agent. In the in-silico study stigmasterol exhibits the strongest binding affinity to the Akt1 protein, with a score of -10.60 kcal/mol (Li et al. 2021). These findings suggest that stigmasterol can effectively target tumour endothelial cells by inhibiting the expression of Akt1, potentially inhibiting tumour development.

Stigmasterol, isolated from *Typhonium flagelliforme*, exhibits a strong binding affinity with farnesoid X receptor (FXR) (Sianipar et al. 2021). With a binding energy of -12.01 kcal/mol. FXR is considered a potential therapeutic target for various liver disorders, including primary biliary cholangitis and primary sclerosing cholangitis (Jiang et al. 2021). The interaction of stigmasterol with FXR could contribute to developing novel therapeutic strategies for treating these diseases. β-Stigmasterol, responsible for antivenom activity against the abundant PLA2 protein in Viper (*Daboia russelii*) and Cobra (*Naja naja*) venom, was identified and characterised from the leaf extract of *Pittosporum dasycaulon* (Chakkinga Thodi et al. 2023). In-silico analyses revealed that the β-SS-PLA2 *D. russelii* complex exhibited a stable conformation with a high-affinity binding energy of -10.60 kcal/mol throughout the simulation period. In contrast, the β-SS-PLA2 *N. naja* complex showed a binding energy of -10.39 kcal/mol. These results suggest that β-stigmasterol has a stronger inhibitory effect against *D. russelii* venom and could serve as an effective antivenom agent.

The stigmasterol components found in *B. trixago* plants exhibit strong binding affinity with the Complex III2 structure of *C. albicans* (-8.7 kcal/mol), the *E. coli* toxin-antitoxin system HipBST (-7.2 kcal/mol), and the large ribosomal subunit of *S. aureus* (-7.0 kcal/mol).

The Rieske head domain in Complex III2 from *Candida albicans* refers to the Rieske head region within the Complex III2 structure of *C. albicans*. Complex III2 is a part of the mitochondrial respiratory chain, involved in electron transfer. The Rieske head domain is a crucial component of this complex, typically containing iron-sulfur (Fe-S) clusters, which are essential for electron transfer. This region plays a key role in the catalytic functions of the complex and is involved in oxygen transport and energy production processes. The binding of the stigmasterol ligand to the Rieske head region within the Complex III2 structure could inhibit fungal CIII2 (Chakkinga Thodi et al. 2023).

HipBST activates the HipT toxin by targeting tryptophanyl-tRNA synthetase (TrpS), and the activity of this toxin is inhibited by the HipS antitoxin. HipS is homologous to the N-terminal subdomain of HipA and inhibits kinase activity by inserting a conserved Trp residue into the active site. By similarly binding to these proteins, it can be suggested that stigmasterol may enhance the inhibition of HipT or modulate the activity of HipS. In this context, stigmasterol is thought to potentially increase its antibacterial activity by affecting the function of the HipBST system (Bærentsen et al. 2022).

The crystal structure of the *S. aureus* ribosome and the binding mechanisms of antibiotics such as linezolid, telithromycin, and a new pleuromutilin derivative, BC-3205, have been studied. Similarly, stigmasterol may interact with ribosomal structures, potentially enhancing the effectiveness of antibiotics or making existing antibiotics more effective against resistant bacteria. Specifically, identifying unique motifs in the ribosomal structure could serve as a target for improving the selectivity and efficacy of antibiotics by binding to these motifs. In this context, it is suggested that stigmasterol could create a synergistic effect with antibiotics or provide enhanced activity against resistant bacterial strains (Eyal et al. 2015).

In conclusion, molecular docking studies emphasise the potential of stigmasterol as a promising therapeutic agent in various biomedical applications. Its strong binding affinity to key proteins such as 4FDO, AKT1, and FXR suggests that stigmasterol could target *M. tuberculosis*, inhibit tumour development, and offer new treatment strategies for liver diseases. Furthermore, the antivenom activity of β-stigmasterol against venom proteins from *D. russelii* and *N. naja* supports its potential as an effective agent for toxin neutralisation. Additionally, stigmasterol’s interactions with fungal and bacterial components, such as *C. albicans* Complex III2, the HipBST system, and the ribosomal subunit of *S. aureus*, suggest that it could enhance the efficacy of existing antibiotics and provide novel strategies against antimicrobial resistance. These findings highlight stigmasterol’s significant promise in drug development, particularly for tuberculosis, cancer, liver disorders, and antimicrobial resistance.

### Molecular properties and pharmacokinetics of stigmasterol

These findings indicate that stigmasterol forms favourable interactions with critical residues within the active sites of these proteins. This suggests its potential to modulate protein functions in bacterial and fungal systems, including influencing bacterial ribosomal activities.

The molecular properties of stigmasterol are as follows: It has a molecular weight of 412.69 g/mol and contains 30 heavy atoms. The number of aromatic heavy atoms is 0, indicating a non-aromatic structure. The fraction Csp3 is 0.86, suggesting a high proportion of sp3 hybridised carbons in the molecule. It has 5 rotatable bonds. There are 1 hydrogen bond acceptor and 1 hydrogen bond donor. The molar refractivity is 132.75, and the Topological Polar Surface Area (TPSA) is 20.23 Å² (Table 6). These properties provide significant insights into stigmasterol’s structural characteristics and potential interactions in biological systems.

**Table 5:** Docking scores and report of predicted interactions of docked conformations of compounds against Escherichia *coli* toxin-antitoxin system HipBST (7AB4), Complex III2 from *Candida albicans*, inhibitor free, Rieske head domain in c position (7RJD) and the crystal structure of the large ribosomal subunit of Staphylococcus aureus (4WCE)

Ligand	Protein	Binding Energy (kcal/mol)	Amino acids	Interacting	Distance
Stigmasterol	7RJD	-8.7	K: SER34: HA	Carbon Hydrogen Bond	2.40
			K: LEU198	Alkyl	5.33
			HEM402	Alkyl	5.00
			HEM402:C9	Alkyl	3.41
			C10- K: LEU17	Alkyl	4.82
			C10- K: LEU198	Alkyl	4.32
			C16- K: LEU198	Alkyl	3.79
			C16- K: LEU201	Alkyl	3.91
			C20- K: MET221	Alkyl	4.53
			C20-: HEM402	Alkyl	4.72
			K: TYR16-:C10	Pi-Alkyl	5.30
			K: TYR16-:C16	Pi-Alkyl	4.51
	7AB4	-7.2	H4-F: GLN151:OE1	Conventional Hydrogen Bond	2.01
			E: PRO62	Alkyl	5.29
			E: LEU93	Alkyl	4.80
			C6- E: LEU60	Alkyl	4.43
			C10-F: ARG214	Alkyl	3.98
			C16- F: LYS64	Alkyl	5.00
			C20- E: PRO62	Alkyl	4.39
	4WCE	-7.0	H4- B: LYS123:O	Conventional Hydrogen Bond	1.73
			B: GLY175:HA2-: O1	Carbon Hydrogen Bond	2.61
			C6- B: VAL121	Alkyl	5.05
			C16- B: ARG172	Alkyl	3.78
			H4- B: LYS123:O	Conventional Hydrogen Bond	1.73

The findings on the pharmacokinetic properties of stigmasterol indicate limited absorption from the digestive system, inability to cross the blood-brain barrier, and non-substrate status for P-glycoprotein. Additionally, stigmasterol exhibits inhibition capability towards the CYP2C9 enzyme but does not inhibit CYP1A2, CYP2C19, CYP2D6, or CYP3A4 enzymes. Its skin permeability is low (-2.74 cm/s) (Table 7). These characteristics may be significant in stigmasterol’s potential biological effects and pharmacological applications.

**Table 6:** Physicochemical properties of the phytoconstituents

Properties	Stigmasterol
Molecular weight [g/mol]	412.69
Num. heavy atoms	30
Num. arom. heavy atoms	0
Fraction Csp3	0.86
Num. rotatable bonds	5
Num. H-bond acceptors	1
Num. H-bond donors	1
Molar refractivity	132.75
TPSA [Å^2^]	20.23

Stigmasterol demonstrates favourable safety profiles across various toxicity assessments. It shows no mutagenic properties in the AMES test. The maximum tolerated dose in humans is determined to be -0.664 log mg/kg/day. While it does not inhibit hERG I channel, it does inhibit hERG II channels. The oral LD_50_ for acute toxicity in rats is 2.54 mol/kg, and the chronic toxicity LOAEL is 0.872 log mg/kg_bw/day. Stigmasterol does not exhibit hepatotoxic effects or sensitise the skin. Its toxicity to *T.Pyriformis* is measured at 0.443 log ug/L, and to minnows at -1.675 log mM (Table 8). These findings indicate that stigmasterol is generally safe, showing moderate acute toxicity in rat models alongside specific hERG II channel inhibition. Its non-hepatotoxic and non-sensitising properties enhance its potential suitability for various applications.

**Table 7:** Pharmacokinetics parameters of the phytoconstituent

Properties	Stigmasterol
GI absorption	Low
BBB permeant	No
P-gp substrate	No
CYP1A2 inhibitor	No
CYP2C19 inhibitor	No
CYP2C9 inhibitor	Yes
CYP2D6 inhibitor	No
CYP3A4 inhibitor	No
Log Kp (skin permeation)	-2.74 cm/s

**Table 8:** ADMET and toxicity profile

Properties	Stigmasterol
AMES toxicity	No
Max. tolerated dose (human) (log mg/kg/day)	-0.664
hERG I inhibitor	No
hERG II inhibitor	Yes
Oral Rat Acute Toxicity (LD50) (mol/kg)	2.54
Oral Rat Chronic Toxicity (LOAEL) (log mg/kg_bw/day)	0.872
Hepatotoxicity	No
Skin Sensitisation	No
*T.Pyriformis* toxicity(log ug/L)	0.443
Minnow toxicity (log mM)	-1.675

## Conclusion

Our study extensively evaluated the chemical composition and biological activities of ethanol and methanol extracts from *B. trixago* flowers. The findings demonstrate significant antioxidant and antimicrobial activities of the extracts. Methanol extract showed lower IC_50_ values in DPPH and iron chelation tests, indicating higher antioxidant activity. High levels of total phenolic and flavonoid contents were detected in both extracts, supporting the presence of compounds with strong antioxidant properties. Antimicrobial activity was tested against various Gram-positive and Gram-negative bacteria and yeast strains. Both extracts exhibited potent antimicrobial effects, with the highest activity against *K. pneumoniae* strains. This suggests that *B. trixago* extracts could provide alternative antimicrobial agents amid antibiotic resistance. Compounds of stigmasterol found in *B. trixago* plants showed high binding affinity to target proteins such as *E. col*i’s toxin-antitoxin system HipBST (7AB4), *C. albicans’* Complex III2 structure (7RJD), and *S. aureus’* large ribosomal subunit (4WCE). These results highlight the potential use of these compounds as antibiotic candidates, representing an essential step in developing new antimicrobial treatment options. ADMET analysis of stigmasterol revealed promising pharmacokinetic properties alongside a safe profile. The findings of this study underscore the potential of *B. trixago* as a valuable source of natural antioxidants and antimicrobial agents.

The antioxidant and antimicrobial activities of *B. trixago* support its pharmaceutical potential. Notably, active compounds such as stigmasterol exhibit strong biological binding affinities. In the future, conducting more comprehensive comparative analyses of the chemical composition and biological activity of *B. trixago* samples from different geographical regions will allow for a broader evaluation of this plant’s therapeutic applications. Furthermore, verifying the efficacy of stigmasterol and other active compounds through in vitro and in vivo models and investigating their effectiveness against resistant bacteria in antibiotic development studies will be a crucial step toward potential medical applications.

## Electronic supplementary material

Below is the link to the electronic supplementary material.

Supplementary Material 1

## Data Availability

All data generated or analyzed during this study are available from the corresponding author upon reasonable request.
