# Supplementary material for: Phytochemical, antioxidant, and antimicrobial properties of Bellardia trixago methanol and ethanol extracts: insights from ADMET and molecular docking approaches
Source: J Food Sci Technol. 2025 Jan 27;63(4):647–63. doi: 10.1007/s13197-025-06217-y (PMC13013873; doi:10.1007/s13197-025-06217-y)
Supplement: Supplementary file 1 — Supplementary Material 1 [file 13197_2025_6217_MOESM1_ESM.docx]

**Supplementary Material Figure**


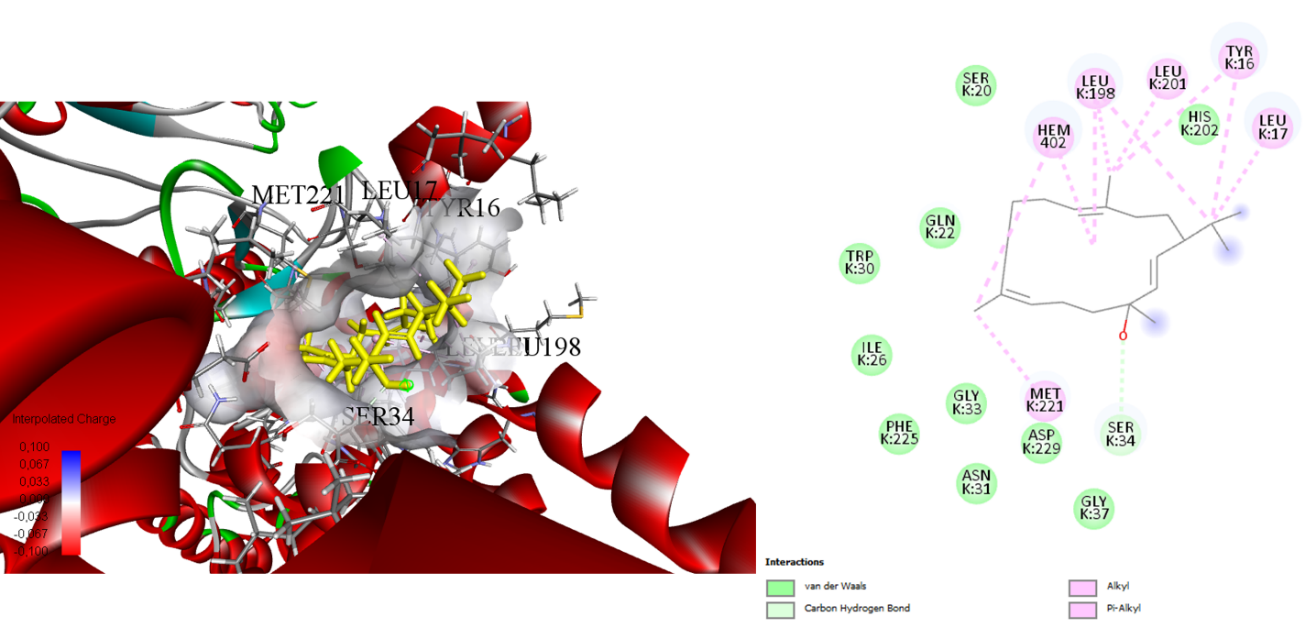


**Figure 2**. Molecular docking process Stigmasterol with Complex III2 from *C. albicans*, inhibitor free, Rieske head domain in c position (7RJD)

**
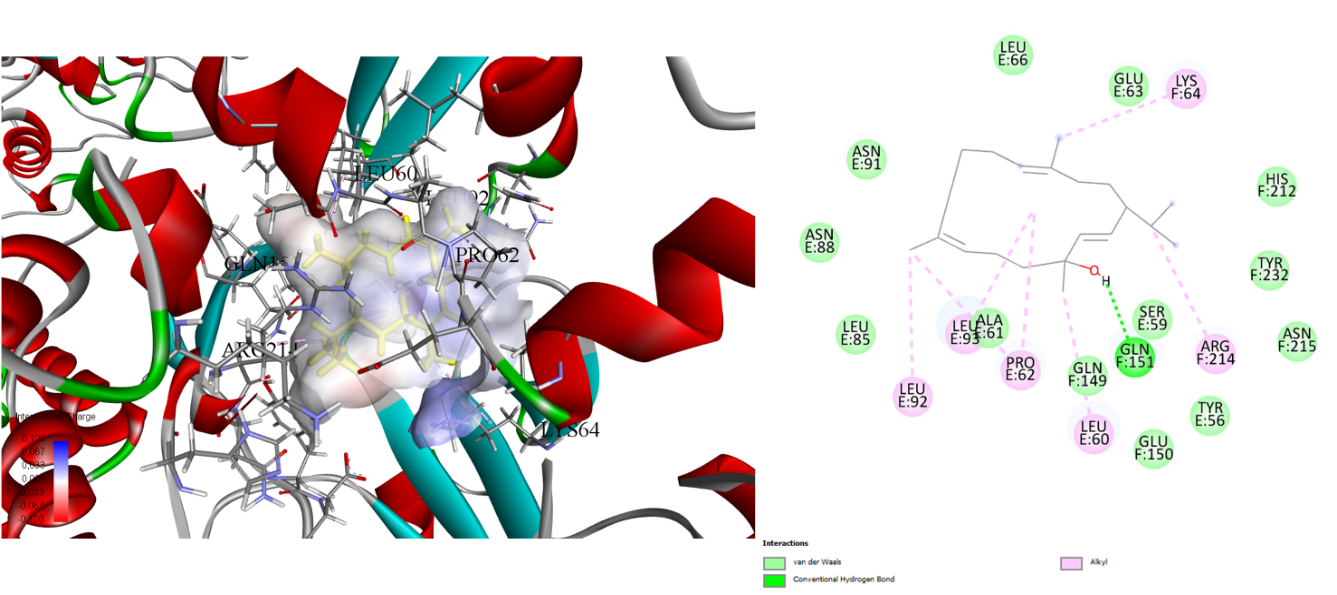
**

**Figure 3**. Molecular docking process Stigmasterol with *E. coli* toxin-antitoxin system HipBST (7AB4)


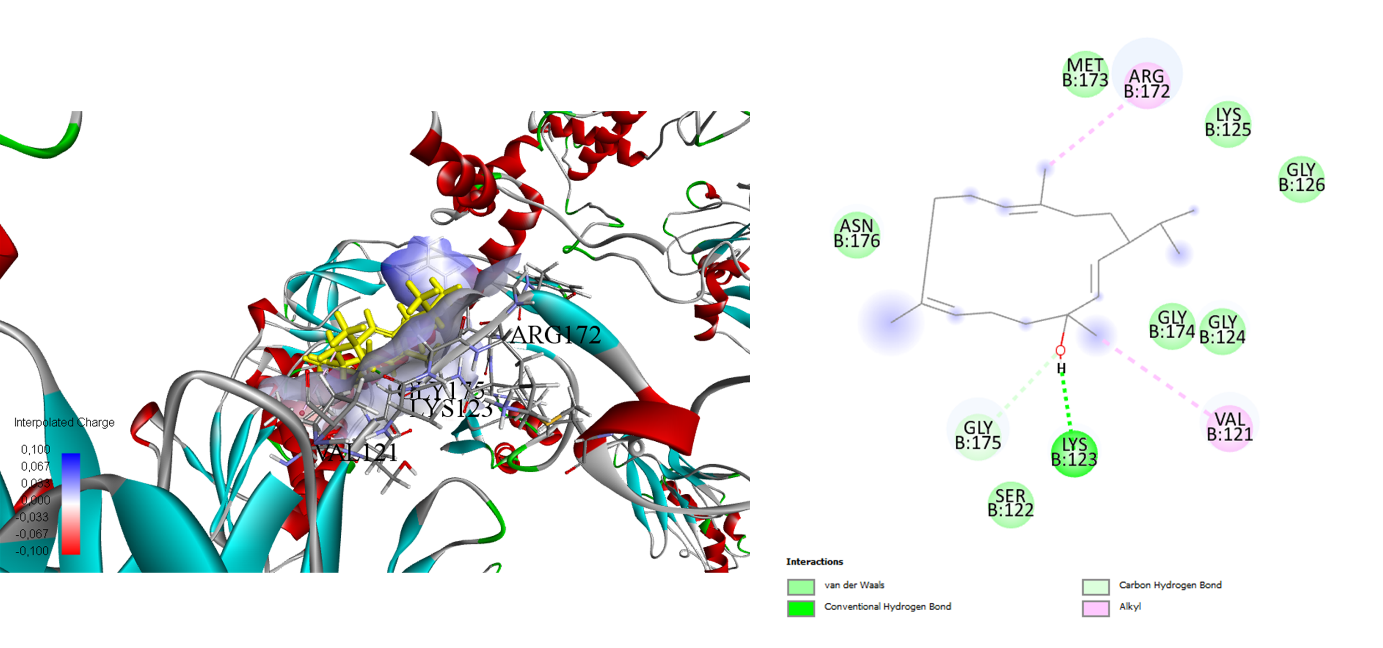


**Fig 4**. Molecular docking process Stigmasterol with the large ribosomal subunit of *S. aureus* (4WCE)

**Supplementary Material Table**

**Table 5** Docking scores and report of predicted interactions of docked conformations of compounds against Escherichia *coli* toxin-antitoxin system HipBST (7AB4), Complex III2 from *Candida albicans*, inhibitor free, Rieske head domain in c position (7RJD) and the crystal structure of the large ribosomal subunit of *Staphylococcus aureus* (4WCE).

| Ligand | Protein | Binding Energy  (kcal/mol) | Amino acids | Interacting | Distance |
| --- | --- | --- | --- | --- | --- |
| Stigmasterol | 7RJD | -8.7 | K: SER34: HA | Carbon Hydrogen Bond | 2.40 |
|  |  |  | K: LEU198 | Alkyl | 5.33 |
|  |  |  | HEM402 | Alkyl | 5.00 |
|  |  |  | HEM402:C9 | Alkyl | 3.41 |
|  |  |  | C10- K: LEU17 | Alkyl | 4.82 |
|  |  |  | C10- K: LEU198 | Alkyl | 4.32 |
|  |  |  | C16- K: LEU198 | Alkyl | 3.79 |
|  |  |  | C16- K: LEU201 | Alkyl | 3.91 |
|  |  |  | C20- K: MET221 | Alkyl | 4.53 |
|  |  |  | C20-: HEM402 | Alkyl | 4.72 |
|  |  |  | K: TYR16-:C10 | Pi-Alkyl | 5.30 |
|  |  |  | K: TYR16-:C16 | Pi-Alkyl | 4.51 |
|  | 7AB4 | -7.2 | H4-F: GLN151:OE1 | Conventional Hydrogen Bond | 2.01 |
|  |  |  | E:PRO62 | Alkyl | 5.29 |
|  |  |  | E: LEU93 | Alkyl | 4.80 |
|  |  |  | C6- E: LEU60 | Alkyl | 4.43 |
|  |  |  | C10-F: ARG214 | Alkyl | 3.98 |
|  |  |  | C16- F: LYS64 | Alkyl | 5.00 |
|  |  |  | C20- E:PRO62 | Alkyl | 4.39 |
|  | 4WCE | -7.0 | H4- B: LYS123:O | Conventional Hydrogen Bond | 1.73 |
|  |  |  | B: GLY175:HA2-: O1 | Carbon Hydrogen Bond | 2.61 |
|  |  |  | C6- B: VAL121 | Alkyl | 5.05 |
|  |  |  | C16- B: ARG172 | Alkyl | 3.78 |
|  |  |  | H4- B: LYS123:O | Conventional Hydrogen Bond | 1.73 |

**Table 6** Physicochemical properties of the phytoconstituents

| **Properties** | **Stigmasterol** |
| --- | --- |
| Molecular weight [g/mol] | 412.69 |
| Num. heavy atoms | 30 |
| Num. arom. heavy atoms | 0 |
| Fraction Csp3 | 0.86 |
| Num. rotatable bonds | 5 |
| Num. H-bond acceptors | 1 |
| Num. H-bond donors | 1 |
| Molar refractivity | 132.75 |
| TPSA [Å^2^] | 20.23 |

**Table 7** Pharmacokinetics parameters of the phytoconstituent

| Properties | **Stigmasterol** |
| --- | --- |
| GI absorption | Low |
| BBB permeant | No |
| P-gp substrate | No |
| CYP1A2 inhibitor | No |
| CYP2C19 inhibitor | No |
| CYP2C9 inhibitor | Yes |
| CYP2D6 inhibitor | No |
| CYP3A4 inhibitor | No |
| Log Kp (skin permeation) | -2.74 cm/s |

**Table 8** ADME and toxicity profile

| **Properties** | **Stigmasterol** |
| --- | --- |
| AMES toxicity | No |
| Max. tolerated dose (human) (log mg/kg/day) | -0.664 |
| hERG I inhibitor | No |
| hERG II inhibitor | Yes |
| Oral Rat Acute Toxicity (LD50) (mol/kg) | 2.54 |
| Oral Rat Chronic Toxicity (LOAEL) (log mg/kg_bw/day) | 0.872 |
| Hepatotoxicity | No |
| Skin Sensitisation | No |
| *T.Pyriformis* toxicity(log ug/L) | 0.443 |
| Minnow toxicity (log mM) | -1.675 |
